# Effect of Number of Tests on the Mechanical Characteristics of *Agave sisalana* Yarns for Composites Structures: Statistical Approach

**DOI:** 10.3390/polym15132885

**Published:** 2023-06-29

**Authors:** Mounir Gahgah, Ahmed Belaadi, Messaouda Boumaaza, Hassan Alshahrani, Mohammad K. A. Khan

**Affiliations:** 1 Department of Mechanical Engineering, Faculty of Technology, University 20 Août 1955-Skikda, El-Hadaiek Skikda 21000, Algeria; 2 Laboratory of Civil and Engineering Hydraulic (LGCH), University 8 Mai 1945 Guelma, Guelma 24000, Algeria; 3 Department of Mechanical Engineering, College of Engineering, Najran University, Najran P.O. Box 1988, Saudi Arabia; 4 Scientific and Engineering Research Centre, Deanship of Scientific Research, Najran University, Najran P.O. Box 1988, Saudi Arabia

**Keywords:** sisal fiber, yarn, mechanical testing, hazard function, statistical methods, SEM

## Abstract

A designer of sustainable biocomposite structures and natural ropes needs to have a high confidence interval (95% CI) for mechanical characteristics data of performance materials, yet qualities for plant-based fibers are very diverse. A comprehensive study of the elements that enhance the performance of biocomposites or sustainable ropes created from vegetable fibers is necessary. The current study included five groups with varying numbers (N) of tests of 20, 40, 60, 80, and 100 on the mechanical characteristics at room temperatures. The purpose of this study was to determine how changing N affects the mechanical properties of sisal yarn. These properties include its strength, Young’s modulus, and deformation at rupture. A significance testing program including more than 100 tests was performed. Owing to the heterogeneity of the plant yarn, each group received more than 20 samples at a gauge length (GL) of 100 mm. The tensile strength characteristics of sisal yarns produced a wide range of findings, as is common for natural fibers, necessitating a statistical analysis. Its dispersion was explored and measured using the statistical methods. The Weibull distribution with two parameters and a prediction model with a 95% confidence level for maximum likelihood (ML) and least squares (LS) were used to investigate and quantify its dispersion.

## 1. Introduction

Due to their potential to be recycled and biodegraded, ecological composites reinforced with fibers are becoming more and more popular as substitutes for synthetic composite materials in a variety of applications [1]. Sisal fibers, the toughest natural fibers, are increasingly used in advanced materials such as composites to lessen the material’s overall environmental effect [2]. High-performance composites made from renewable sources are becoming increasingly popular in the composites industry and among its consumers. Because of their exceptional formability, these composites are frequently manufactured using yarn-based textile engineering [3,4]. However, to use natural thread as a reinforcement system in structural composite applications that are highly loaded, its mechanical performance must be significantly improved [5,6].

Several studies are now being conducted to better understand the fundamental qualities of natural fibers, such as high-quality biocomposite raw materials [7,8]. These studies are critical for establishing their intended ideal usage. There is also a discussion about how to improve the longevity and commercial worth of natural fibers through the study and characterization of their qualities and potential applications. Natural fibers have a low density and high strength-to-weight ratio, making them good candidates for lightweight composites and reinforcing materials [9]. The chemical composition and microstructure of fibers have an impact on their mechanical properties, with the fiber cross section being the most influential variable on fiber strength [10]. Moreover, the anatomical qualities of fibers differ between species, influencing their density and mechanical properties. Various factors, such as fiber extraction, storage duration, and environmental conditions, affect the size and quality of natural fibers [7,11,12].

The mechanical characteristics using vegetable sisal yarns exposed to tension quasi-static stress with a 13° twist angle and 232 tex linear density were examined by Belaadi et al. [13]. A test procedure was performed on 150 samples. Because natural yarns vary in quality, more than 30 specimens were evaluated for each GL. Five distinct GLs (from 50 mm to 300 mm) were used. The findings obtained were then analyzed utilizing statistical Weibull distributions with two and three parameters for various probability indexes, and LS and ML estimates. The author showed that as the GL increases, the strain and tension on the yarn drop from 14.7% to 5% and 180 MPa to 122 MPa, respectively. Nevertheless, Young’s modulus is unaffected by the GL. A recent study by Belaadi et al. [14] was taken as the basis to examine the behavior of 30 samples of jute yarn with a GL of 150 mm, an area angle torsion of 10° to 13°, a linearly distributed density of 27.021 tex (g/1000 m), and an average diameter of 500–1100 m. Based on the data of the tested fibers, we found the mean strength to be 91.69 ± 21.38 MPa, the average strain to be 2.18 ± 0.34%, and the Young’s modulus to be roughly 3163 ± 994 MPa. These numbers (Young’s modulus and strength) show an increase of almost 14.20% and 22.5%, respectively, compared to the results found by Codispoti et al. [15] for jute yarn with a gauge length of 100 mm. Another novel approach was employed by El-Geiheini et al. [16] to enhance the mechanical properties of cotton yarns. The resulting models were developed by applying artificial neural network techniques to processed images.

Yan et al. [17] used a 150 mm length soaked for 30 min in a solution of 5 wt% NaOH to test the tensile strength of three distinct types of processed bamboo, flax, and linen threads. Ten iterations of each test were conducted. The tensile failure stress of unprocessed flax single-strand yarn is 115.4% higher than that of unprocessed bamboo and 12.1% higher than that of unprocessed linen. Linen yarns have over 50% greater elongation at the break point than flax and bamboo yarns. All three fiber yarns’ tensile strengths and moduli were lower compared to their alkali-treated counterparts. When compared to untreated specimens, potassium hydroxide-treated linen, flax, and bamboo strings had lower tensile strengths and tensile moduli of 16.4%, 18.5%, and 30.7% and 13.0%, 5.9%, and 27.8%, respectively.

On the other hand, alkali-treated linen and flax yarns had greater elongations at rupture. Gomes et al. [18] achieved a comparable outcome in the case of a curaua fiber with alkali treatment. Saaidia et al. [19] discussed the statistical characterization of the tensile characteristics of 13 series of 30 samples of raw and treated jute yarns with twisted surface angles ranging between 11° and 13°, with a linear density of 267 tex at GL = 50 mm. The yarns were treated with various NaOH concentrations and immersion periods. Weibull techniques with two and three parameters were used for the statistical analysis. The findings for uniaxial tensile yarns reveal a variance in terms of failure strain, stress, and Young’s modulus, which is mostly determined by the period of immersion and the concentration of NaOH. An immersion duration of two hours with a NaOH dosage of 2% produced the best mechanical characteristics.

Abida et al. [20] conducted an experiment examination on the morphological and mechanical characteristics of yarns of flax, revealing the three characteristic distributions to be normal distributions. The suggested model implies that tissue is made up of many threads that work together like springs, and linen threads are fragile and flexible. A genetic algorithm with multiobjective analysis was utilized to solve the inverse issue to identify the yarn characteristics from experimental tensile testing. Abida et al. [21] conducted a new study on the coefficients of hygroscopic expansion of flax fiber- and flax thread-strengthened composites along three orientations using a combined experimental multiscale approach. This study found that flax yarn had a significant radial coefficient of expansion (β_r_ = 1.06), and the composite showed strongly anisotropic bulking behavior. In addition, the weft direction exhibited good dimensional stability: β_r_ = 0.13 ± 0.019. The radial expansion of the warp threads and matrix is balanced by the negative axial hygro-expansion factor in the weft threads, which can be explained by the longitudinal and out-of-plane swelling factors.

Wang et al. [22] developed a statistical model to examine the random tensile response of 30 natural jute fiber yarns at GL = 100 mm, taking fiber crimp and property distribution into account. The statistical properties of corrugated jute fibers are described as a distribution probability beta function fiber strain. Despite the fact that the effective modulus of elasticity and thread strength obey the law of normal distribution, the tension graphs of similar thread specimens exhibit identical form features to the beta dispersion in the crimped strain. This pattern may provide reasonable forecasting restrictions for the dispersion of the jute fiber nonlinear tensile response. Sohbatzadeh et al. [23] presented a new method for the low-cost modification of the synthetic aramid thread surface. In this study, plasma systems at atmospheric pressure were used to surface modify the threads with argon as the working gas and acetone as the precursor. These modifications resulted in improved tension strength and flotation characteristics, with the treated thread displaying superb waterproofing and buoyancy characteristics. In addition, the modifications by plasma enhanced the thread’s mechanical strength and, thus, its suitability for reinforcing applications. Additionally, the study revealed that desirable carbon-based nano-structures were synthesizable on the yarn surface.

Consequently, an evaluation of the mechanical performance using the fibers and threads themselves is necessary for the application of sisal threads as load-bearing cables. The literature has covered a lot of research on synthetic yarn. However, the use of statistical methods to estimate the mechanical performance of natural yarns requires further investigation. In this study, the focus was on the mechanical characterization of yarns using various sample numbers. Two-parameter Weibull statistics for various estimators and techniques (ML and LS predictions) were used to assess the tensile properties. Additionally, a thorough analysis of the study’s findings was conducted and contrasted with information from the literature. To the knowledge of the authors, this represents the first instance in which the yarns have been investigated at various sample sizes using a predictive model with a 95% confidence level.

## 2. Materials and Methods

**Figure 1:**
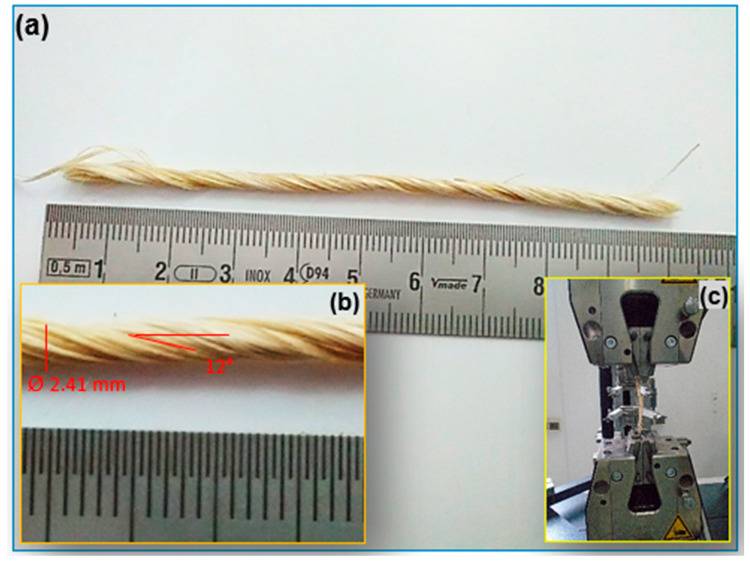
(**a**) Single specimen; (**b**) geometry of the sisal yarn used in this work; and (**c**) specimen clamped.

The yarns made from the *Agave sisalana* utilized in this study were formed of carded sisal fibers, which are particularly suitable for producing yarns and ropes of varying widths. The Algerian packaging and cable company BLIDA provided the yarns. The sisal fiber was 0.76 to 1.4 m long, 248 µm in diameter, and had a cross-sectional area of 0.043 ± 0.008 mm^2^. The mean mechanical parameters of individual separate-carded sisal fibers employed for this investigation, with a density of 1.43 g/cm^3^, were previously published by Belaadi et al. [24] as = 463 MPa, *ε* = 7.84%, and *E* = 7.39 GPa. The sisal yarns were made up of about 70–80 sisal fibers with a diameter of approximately 2.41 ± 0.64 mm and a cross-sectional area of 4.52 ± 0.43 mm^2^. In addition, the sisal yarns (Figure 1a,b) had a linear density of 223 ± 42 tex (g/1000 m) with a mean twisting angle of approximately 10–13° (Table 1). The diameter of each sisal thread was measured using a ZEISS microscope instrument fitted with a Moticam 2500 camera that was digitally controlled by the Motic-Images Pro V2.0 visualization software. Over its entire length, the diameter changed. Ten measurements were performed at various points along the yarn. Using the mean yarn diameter, the observable cross-sectional measurement for each yarn was computed. In addition, the fiber cross section was assumed to be circular with a constant diameter throughout its length [13,22].

Minimum values of 20 basic sisal yarns were used to determine the tensile mechanical behavior characteristics (the modulus of elasticity, ultimate elongation, and tensile strength) for five groups (N) of 20, 40, 60, 80, and 100 in accordance with ASTM D2256-01 [25]. The sisal yarn samples were selected to be a mean length of 100 mm. The latter were carefully handled to avoid deterioration. The Zwick-Roell testing apparatus with a 50 kN load cell and mechanical grips was used for all of the experiments (Figure 1c) at GL = 100 mm. At a steady rate of 5 mm/min, tensile tests were carried out in the laboratory at a temperature of 25 °C and a relative humidity of 40%.

**Figure 2:**
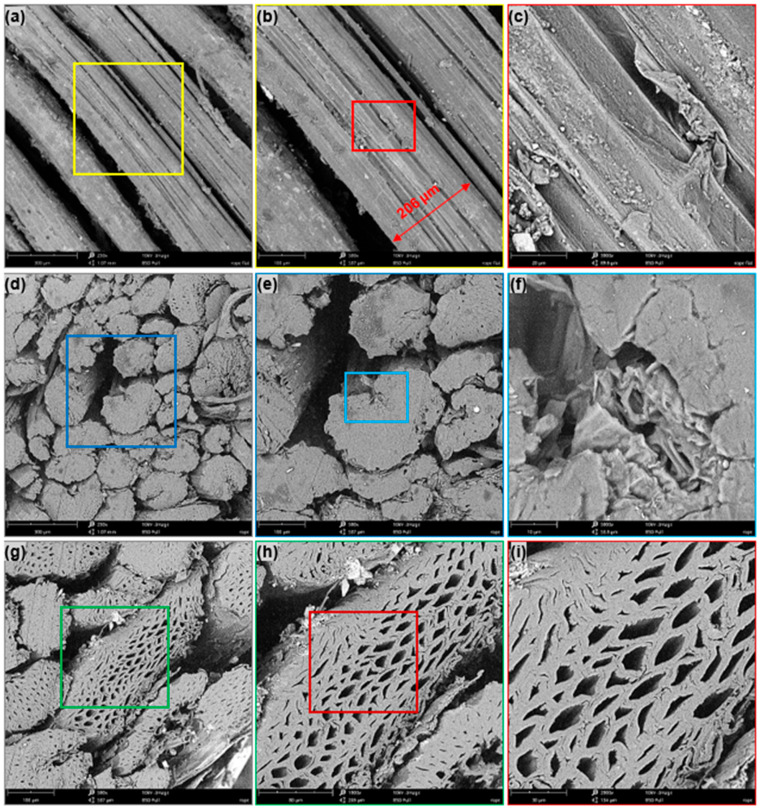
SEM micrographs of (**a**) longitudinal view of sisal yarn, (**b**) diameter measurements for single sisal fiber, (**c**) topographic surface of sisal fiber, (**d**) cross-sectional view of sisal yarn, (**e**,**f**) zoomed details of the selected zone in (**d**,**g**) another view of the cross section of the same yarn from (**d**), and (**h**,**i**) zoomed details of the selected zone in g showing the crushing of sisal fiber cells.

An individual sisal fiber, thread, and sections were examined using a JSM-7600F scanning electron microscopy (SEM). A thin layer of gold was applied to make the samples conductive. At an accelerating voltage, SEM micrographs were captured (Figure 2a–i).

## 3. Weibull Statistics for Sisal Yarns Data

According to the literature, most natural fibers exhibit significant dispersion in their mechanical characteristics. Such scattering can be described by the concentration of imperfections on the surface and the microstructure of the fiber [26,27]. Two- and three-parameter models were used to statistically examine the values of the sisal yarns’ tensile mechanical properties (*σ*, *ε*, and *E*).

To describe the degree of variable tensile properties, the Weibull distribution using different approaches was determined using ML and LS for a confidence level of 95%. Moreover, using mechanical characteristic data, Anderson–Darling (AD) testing with modified quality-of-fit estimations was employed to identify the best fit. Minitab V-16 was used to conduct the statistical analysis. The Laplace–Gauss normal distribution is a rule of absolutely continuous probability, which is determined by two parameters: the standard deviation (SD) and the mean (t and α^2^ is the variance). The parameter (*μ*) provides information about the distribution center. The parameter *s* indicates the extent to which it has spread. Equation (1) [28] gives the probability density function (PDF). The variable $y=\left(x-\mu \right)/\alpha$ is the reduced-centred Gaussian probability density determined using Equation (2) for the variable *μ* = 0 and *α =* 1. The log-normal distribution, on the other hand, is characterized using two parameters: ξ and λ, with λ>0, and one obtained for the PDF (Equation (3)). Equation (4) defines the PDF of the three-parameter Weibull distribution, often known as the failure distribution, or as the cumulative distribution function (CDF). In addition, Equation (5) yields the probability density for *s*_0_ = 0 [22]. By simplifying Equation (5), we obtained a Weibull survival probability with two parameters by assuming *s*_0_ = 0 (*s*_0_ stands for a threshold or localization parameter), which denotes a mean parameter value of *x* (minimum survival time) that can be characterized by Equation (6) [22,29]. Where *m* and *s* represent real positive numbers that represent the shape’s factor such as The Weibull modulus and the scale parameter or characteristic value, respectively.

(1) $$P\left(x|\mu ,\alpha \right)=\frac{1}{\sqrt{2\pi s}}e^{-\frac{1}{2}{\left(\frac{x-\mu }{\alpha }\right)}^{2}}$$

(2) $$\varphi \left(y\right)=\frac{1}{\sqrt{2\pi }}e^{-\frac{y^{2}}{2}}$$

(3) $$P\left(x|\xi ,\lambda \right)=\frac{1}{x\xi \sqrt{2\pi }}e^{-\frac{1}{2}{\left(\frac{\ln\left(x\right)\;-\;\lambda }{\xi }\right)}^{2}}$$

(4) $$F\left(x|s_{0},s,m\right)=1-e^{\left[-{\left(\frac{x\;-\;s_{0}}{s}\right)}^{m}\right]},\;\;\;x\geq s_{0}$$

(5) $$F\left(x|s,m\right)=\frac{m}{s}{\left(\frac{x}{s}\right)}^{m-1}e^{\left[-{\left(\frac{x}{s}\right)}^{m}\right]}$$

(6) $$F\left(x|s,m\right)=1-e^{\left[-{\left(\frac{x}{s}\right)}^{m}\right]}$$

The ML technique is described by the formula in Equation (7) [30] and is a key approach for estimating the probability density parameters or Weibull probability. This strategy depends on the parameters being selected so as to maximize the probability for the specimen information. Since this method provides estimated parameters that have higher statistical properties, this approach was selected due to its statistical consistency. The advantage of this method is that the ambiguity of the distribution of Weibull parameters with confidence intervals of 95% can be easily and efficiently identified. Another approach, known as the LS estimate using linear regression, (Equation (8)) [31] was applied from member to member using the natural logarithm. The Weibull modulus *m* was determined from the slope of a straight line of the $\ln\left(\ln\left(\frac{1}{1-P}\right)\right)$ vr. $\ln\left(x\right)$. This line’s intercept enabled us to determine s. In our case, distinctive mechanical parameters, such as the characteristic Young’s modulus, strength, and strain at failure, i.e., *E*_0_, *σ*_0_, and *ε*_0_, respectively, were obtained. The biggest issue with this methodology is the estimation of the survival probability (*p*). The *p* value was calculated using an estimator or probability index. $P_{i}=\left(i-\tau \right)/\left(n-\beta \right)$ where τ=0.3, and β=0.4 are the generic forms of the estimator. The probability index was extensively used to evaluate the estimator, as shown in Equation (9) [13]. Additionally, numerous alternatives for estimating the probability index are available in the Minitab program, such as the median rank or Benard index (Equation (9)).

(7) $$\mathrm{Likelihood}\left(m,s|x_{1},\ldots ,x_{2}\right)=\prod_{i=1}^{n}F\left(x_{i}\right)=\prod_{i=1}^{n}\frac{m}{s}{\left(\frac{x_{i}}{s}\right)}^{m-1}e^{\left[-{\left(\frac{x}{s}\right)}^{m}\right]}$$

(8) $$\ln\left(\ln\left(\frac{1}{1-P}\right)\right)=m\;\ln\left(x\right)-m\;\ln\left(s\right)$$

(9) $$P_{i}=\frac{i-0.3}{n+0.4}$$

## 4. Results

### 4.1. Tensile Behavior of an Elementary Sisal Yarn

**Figure 3:**
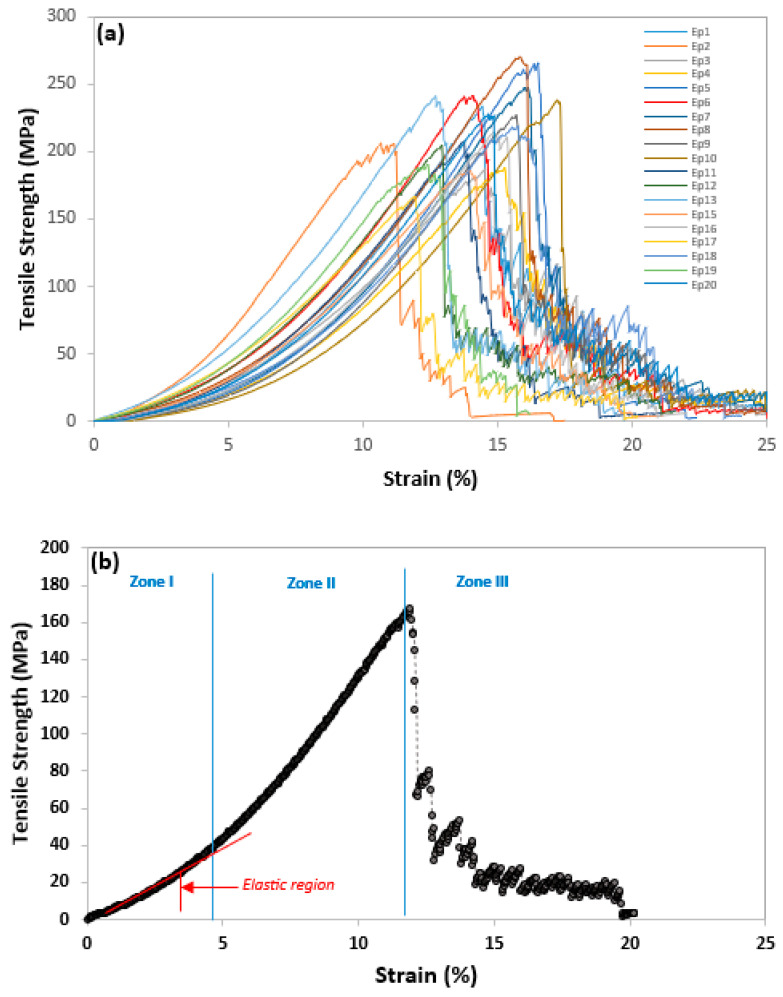
Stress-strain curves (**a**) for first 20 sisal yarns and (**b**) typical behavior for tensile test at GL = 100 mm.

At room temperature, the sisal yarns subjected to tensile tests at a speed of 5 mm/min were divided into five series of 20 specimens, representing a total of 100 elementary yarns that were chosen at random via the specific lot and gauged at GL = 100 mm. The stress–strain graphs from 20 static tensile tests performed on the sisal yarns are displayed in Figure 3a. Clearly, the wide dispersion of results, a phenomenon specific to natural fibers, calls for an in-depth statistical analysis. The same behavior was observed in the case of jute yarn reported by Wang et al. [22] and Saidia et al. [19] at GL = 50 mm, and in the case of sisal yarn [13] and flax yarn [20] in the last 50 samples. As illustrated in Figure 3b, the typical tensile curve behavior of sisal yarns may be characterized into three zones: the crimp, nonlinear, and damage region. The nonlinear region (0–4.5% of strain-*ε*) is most likely related to the rearrangement of elementary fibers in the yarn. In addition, this phenomenon has been explained by several authors [13,28] since sisal yarns are produced by twisting the fibers in a spiral, creating intermediate spaces in the fibers. The Young’s modulus of the threads was also determined using the linear-elastic region (next phase), which had a relatively high slope (5–10% of *ε* and *σ* between 30–170 MPa). The specimen did not completely fail until the stress rapidly decreased to a mean strength of 81 MPa, with 80% of the yarn fibers failing as a result of the third area, i.e., the damage zone.

As a result of 100 tests of sisal yarns at various test numbers (N), the standard deviation (SD) and the mean values of the mechanical characteristics, notably strain at break, stress, and Young’s modulus, are summarized in Table 1. They were analyzed by calculating the percentage of the coefficient of variation (CoV%). The coefficient of variation in percent is defined as the relationship of the mean (*μ*) to the standard deviation (*σ*), as determined through the expression CoV (%) = [(*σ*⁄*μ*) × 100], whereas at a low percentage CoV, it was assumed that there would be little difference in the data. A very high and significant scatter in the values given for tensile properties with various tests N can be seen in Table 2. The tensile strength was determined from the cylindrical assumed cross section of individual threads, with the theoretical area provided by the mean diameter of the filaments. Nevertheless, Young’s modulus was determined in the elastic portion of the stress–strain curve, which is the graph’s initial slope, usually in the range of 0.5 to 1.5% of *ε*. The high degree of scattering observed in the findings is a natural characteristic of yarns related to products with vegetable fibers. In fact, there were several factors contributing to these scatters, which affected the fiber [32,33] and its yarn: (1) Environmental factors that depend on the particular conditions in which the plant fiber was grown, its type, the specific position in the plants, and whether it has an irregular geometry; and (2) experimental factors associated with both the choice of test parameters (speed and precision of deformation, type of machine clamping device, and the environmental conditions in the laboratory (the relative hygrometry influences the behavior of the saw fiber in relation to its hydrophilic character)), and the choice of the measurement geometry (the cross section of the yarn) used in the calculation of the resistance (porosity in percentage). Indeed, it is clear from Table 2 that the number of tests (N = 20, 40, 60, 80, and 100) has a significant impact on the performance of sisal yarns. Thus, in this work, the sisal yarn showed maximum stress values equivalent to 148, 146, 139, 138, and 135 MPa, with a progressive decrease according to the number of tests until N = 80, followed by stabilization until N = 100 tests. The same phenomenon was observed with respect to the values of the failure strain, which also decreased with increasing N: *ε* = 8.41%, 7.83%, 7.37%, 7.15%, and 6.70%. However, when N was increased to 100, a higher Young’s modulus *E* was obtained, i.e., it went from 528 MPa for N = 20, to 660 MPa. Aside from the strength, there was a decrease in the stress of about 82% (148 for N = 20 and 135 MPa for N = 100).

It is difficult to compare the experimental data obtained from the literature (Table 3) given the types of yarn used, its maturity, the environmental conditions in which these plants were grown, and the methods used to perform the tests, especially to determine the rate of stress. Nevertheless, it was possible from the literature [1,5,8,13,15,19,20,22,28,34,35,36,37] to synthesize the results on plant yarns in tensile static tests (Table 3). The strength value obtained from our experiments for sisal yarn equates to 148 MPa for GL = 100 mm in N = 20 tests, which is similar to the reference for N = 30 [13]. Additionally, it shows that, for testing with N = 20, a value of *E* = 528 MPa was obtained, whereas the Young’s modulus for this reference for the same gauge length of 100 mm was 556 MPa. These results are near to those from this study for N = 100, which is equivalent to 660, for the same GL and N = 100. In contrast to the results of the present investigation, the researchers in [15] discovered lower values for *σ* and *E* for GL 100 mm (*σ* = 31.5 MPa and *E* = 85.2 MPa) for N = 10 to 15 tests. With GL = 100 mm and N = 20 trials, the strain rate of sisal yarn during breakage in this investigation was 8.41%, which is nearly identical to the 8.37% for the same GL with N = 30 found in [13]. On the other hand, a lower result for strain rupture equal to 6.7% was obtained by raising the number of trials to 100.

### 4.2. Statistical Distribution of Sisal Yarn Data

**Figure 4:**
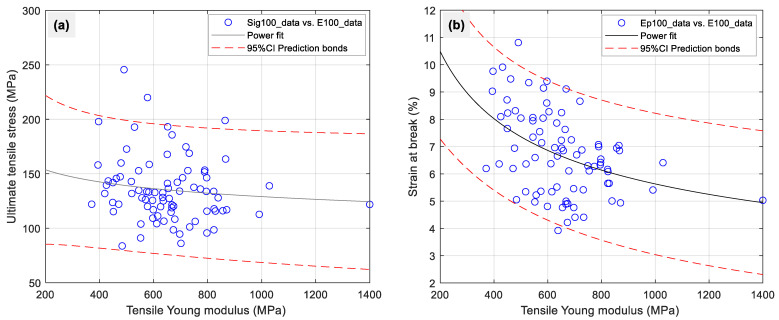
Scatter plots for all 100 tests of sisal yarns for (**a**) tensile strain as function of Young’s modulus and (**b**) tensile strength as a function of Young’s modulus.

The wide variability of mechanical characteristics of cellulosic fibers of plant origin is a challenge for designers of composite structures. To gain a better understanding of the biocomposites benefits or strings produced from plant-based fibers, it is essential to have a good knowledge of the constituents that enhance their properties. Figure 4a,b show the relationship variation between tensile stress and strain at rupture, Young’s modulus, and power fit, with a power prediction model for a 95% CI for different test groups. The Young’s modulus dropped as the strength increased, as shown in the figure, and the relationship between strain at rupture and Young’s modulus also followed this pattern [13].

**Figure 5:**
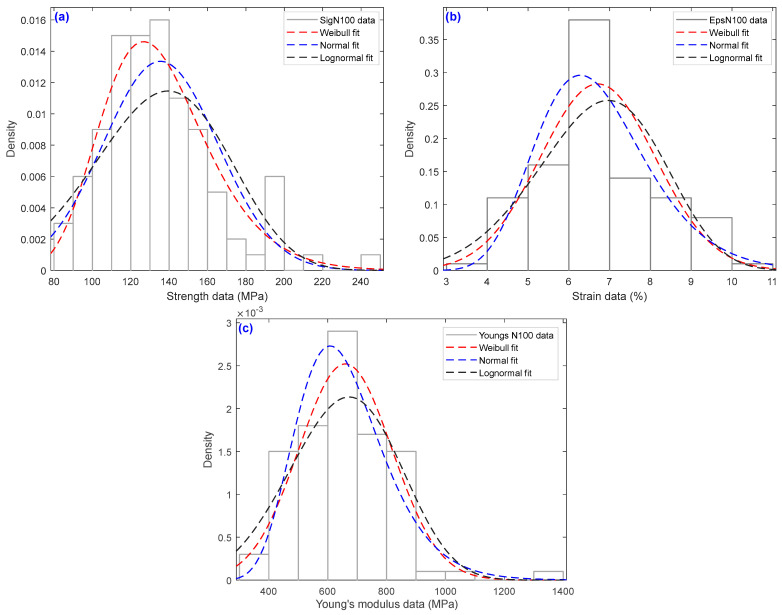
Histograms of the tensile strength data of the sisal yarns at different numbers of tests with the estimation of density functions Weibull, normal, and lognormal for N = 100 (**a**) strength data, (**b**) strain data and (**c**) Young’s modulus data.

Figure 5 illustrates the experimental histograms for the mechanical properties, in particular, *σ*, *ε*, and *E* in the case of N = 100 tests, according to the various distribution methods. To select the cells required for the histogram, we followed the standard square root rule based on the amount of data to be considered. In the field of materials science, various statistical distributions are available, with the Weibull distribution, normal distribution, and lognormal distribution being the ones that are employed the most. Therefore, in materials science, lognormal, Weibull, and normal distributions are frequently used to describe various properties of materials. Indeed, in contrast to the lognormal distribution, which is used to represent the distribution of the size of particles and cracks for materials, the distribution known as the Weibull distribution is frequently employed for strength forecasting and to assess the brittle fracture and reliability of materials. On the other hand, the normal distribution is a versatile distribution that can be used to model a wide range of material properties and describe random phenomena. For mechanical property information, the lognormal distribution or Weibull distribution is typically the most appropriate solution. In this case, to justify this choice, the normality Kolmogorov–Smirnov test (Table 4) was performed and the estimates of the goodness-of-fit with four distinct distributions via the Anderson–Darling test (Table 5) and the AD with *p*-values (Table 6) are presented.

**Figure 6:**
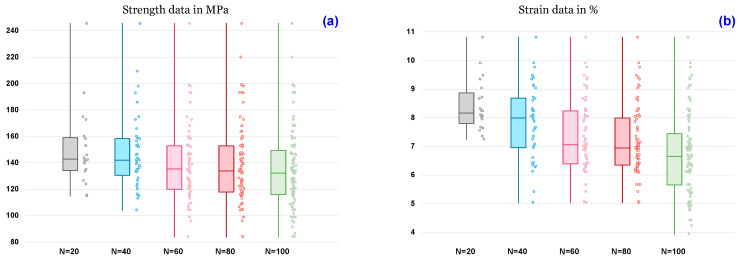

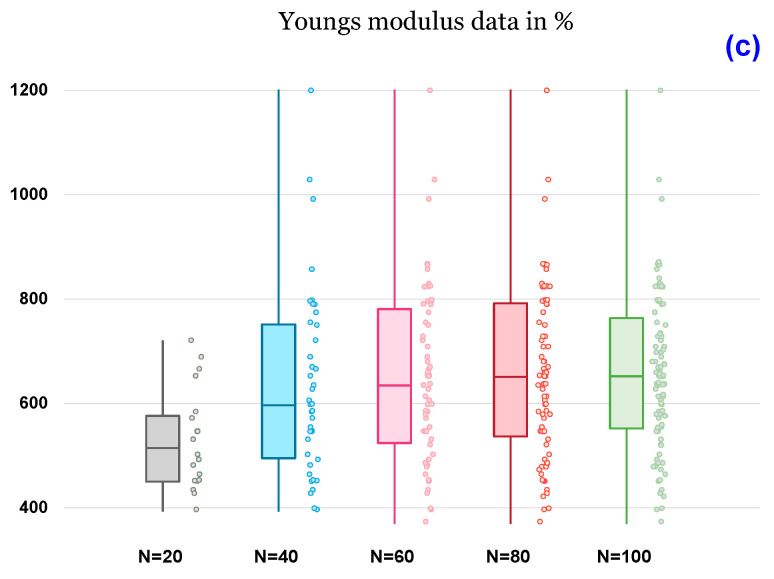
Average tensile properties with the number of tests N for (**a**) strengths data, (**b**) strain at break data, and (**c**) Young’s modulus data.

As a function of the number of specimens, Tukey or box plots were used to display the variance in the average mechanical characteristics of sisal yarns, such as tensile strength, elastic modulus, and strain. (see Figure 6). Box plots were used to show the general trends in the responses of a group. These diagrams are useful for visualizing the distribution and other characteristics of the responses of a large group, such as the mechanical properties. The diagram illustrated in Figure 6 shows a variety of box-plot shapes and positions. This study presents the distribution of data between the number of samples (N) and the mechanical characteristics of sisal yarns with a 95% confidence interval of the forecast. Variable data with maximum, median, minimum, and quartiles (Q1 and Q3) characterize this representation. The extremes are then extended with segments, resulting in extreme values for the first and ninth deciles. For instance, for N = 100, the median, first and third quartiles, and both maximum and minimum numbers of the samples were 131, 118, 148, 245, and 83 MPa for stress, 6.85, 5.85, 7.41, 10.7, and 3.8% for strain, and 651, 551, 763, 1230, and 371 MPa for Young’s modulus. Additionally, it appears that the yarn’s tensile stress (Figure 6a), which followed a power tendency line, decreased as N increased (20–100 tests). Similarly, Figure 5b illustrates the number of samples, N, as a function of elongation at break. It is evident that when N increased, the elongation reduced. On the other hand, as N increased, the mean Young’s modulus rose (Figure 6b). This behavior is similar to that of single Washingtonia fibers, which Dembri et al. [38] observed from 30 to 90 tests before stabilizing at N = 120 and 150 tests.

### 4.3. Normality and Kolmogorov–Smirnov Tests

The test results grouped in Table 4 show that the *p*-value of normality using the Kolmogorov–Smirnov test criteria was >0.15 for elastic modulus data only. Thus, the property dispersion can be described by a 2P Weibull, 3P Weibull, and lognormal distribution. Therefore, it is necessary to determine which of these three laws best describes the experimental results. Additionally, Table 5’s test results demonstrate that the *p*-values for the 2P and 3P Weibull distributions were above 0.1, while the *p*-value for the lognormal distribution was less than 0.1. Although the *p*-value was less than 0.1, in order to exclude a distributional law, the *p*-value recorded for the 3P Weibull law indicates that it was not as effective as the other 2P Weibull laws in describing our data. Thus, the dispersion of the properties can be described using a 2P Weibull distribution.

### 4.4. AD Goodness-of-Fit of Normality of Sisal Yarn Data

Following the Kolmogorov–Smirnov test for normality, we can conclude that the Weibull distribution is the best in terms of describing the behavior of our results. To determine which one of the four proposed distributions (Table 6)—normal, lognormal, 3P-Weibull, or 2P-Weibull—best fits the series (five groups) on which the experimental data was collected, the Minitab software was used to compute and perform an Anderson–Darling (AD) good fit test on every distribution in order to determine the smallest values of AD. This test produces the K-S (Kolmogorov-Smirnov) adjustment. For different numbers of trials (N = 20, 40, 60, 80, and 100 trials), the 2P-Weibull distribution was used to estimate the AD quantities for the sisal yarns Young’s modulus, i.e., AD = 0.837, 0.537, 0.482, 0.664, and 0.626, respectively. These were lower compared to the 3P-Weibull distribution estimates for AD = 0.920, 0.628, 0.452, 0.690, and 0.637. In addition, with respect to strain and stress, the smallest amounts were found for the two-parameter Weibull distribution (strain at break: AD = 0.989, 0.484, 0.543, 0.989, and 0.551 and stress: AD = 0.921, 0.756, 0.475, 0.503, and 0.523). However, the highest values were for the logarithmic distribution, which are given as follows: AD = 3.499, 3.402, 2.784, 3.597, and 4.400 for *σ*; AD = 2.869, 1.662, 2.358, 4.917, and 2.545 for *ε*; and finally, AD = 1.923, 2.883, 1.938, 1.814, and 1.821 for *E*.

### 4.5. Weibull Analysis of Sisal Yarn Data

**Figure 7:**
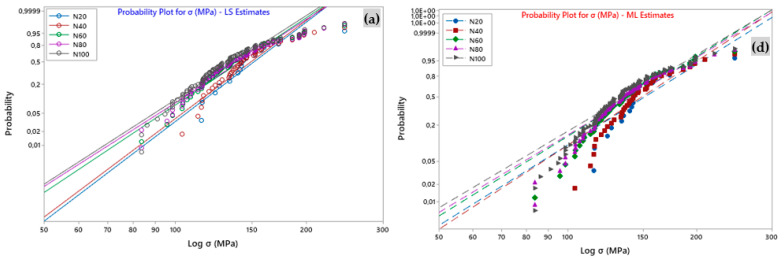

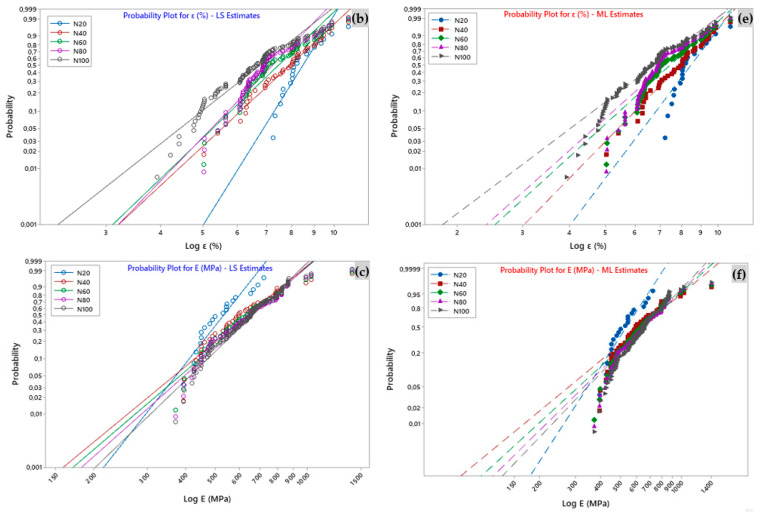
Probability plot for tensile properties with the number of tests N for (**a**–**c**) least square-LS estimation and (**d**–**f**) maximum likelihood-ML estimations.

In this study, the two-parameter Weibull law was used to investigate the data of sisal yarns’ mechanical properties, including the stress, Young’s modulus, and strain, which revealed a significant amount of variation. Thus, Figure 7 well represents the distribution curves according to Weibull-LS and ML, corresponding to the stress, strain, and Young’s modulus of the experimental data. In addition, using the Minitab software, the associated parameters are listed in Table 7. From Figure 7, it can be seen that for different numbers of tests (N), the behavior of the straight lines from the Weibull diagram of two-parameter LS and ML concerning the sisal yarn was almost linear with a superposition and a slight difference between them. However, there was an inflection of the line with respect to the LS and LM Weibull distributions when N = 20 trials with respect to *E* (Figure 7c,f), which resulted in the lowest values. This nonlinearity and almost overlapping behavior was observed at the level of *Agave americana* plant fibers with different GLs, and at the level of sisal elemental fibers [39] and yarn [13] (depending on GL). The R^2^ coefficient is the primary control for evaluating the variation in the Weibull modulus (m). Furthermore, it is worth noting the satisfactory linearity of the fit between all datasets (Figure 7). Indeed, for each estimator, we found a correlation factor (R^2^) of 0.900, 0.930, 0.958, 0.957, and 0.955 for *σ*, 0.910, 0.990, 0.964, 0.946, and 0.972 for *ε*, and 0.944, 0.930, 0.958, 0.964, and 0.969 for the Young’s modulus. In addition, the two-parameter LS-Weibull model provides higher correlations (R^2^ = 0.970–0.990 (Table 7)) with respect to the strain at failure compared to the other features (*σ* and *E*).

Table 7 lists the Weibull distribution’s parameters, forms, and localities (which are its defining values) for each of the mechanical properties. The corresponding Weibull moduli (2P-Weibull-LS) least squares (LS) concerning strain (*m_ε_*), stress (*m_σ_*), and Young’s modulus (*m_E_*) relative to various N at 20, 40, 60, 80, and 100 mm were *m_ε_* = 15.4, 8.7, 6.6, 5.9, 5.2, *m_σ_* = 8.3, 6.4, 5.9, 6.2, and 5.4, and *m_E_* = 1.7, 6.6, 4.8, 4.8, and 5.4, respectively. Consistent with reference [13], the length and amount of testing had a significant impact on the Weibull modulus of the tensile properties of elementary sisal yarns. Thus, for example, at 2P Weibull-LS, the stress moduli (*m_σ_*) were 7.29, 7.21, 6.42, 6.18, and 6.11, respectively, for N = 20 to 100 tests. A similar behavior was observed with respect to all values of the 2P-Weibull modulus (ML) for different values of N (*m_σ_* = 4.91, 4.69, 4.66, 4.52, and 4.47). For comparison (Table 7), the *m_σ_* and *σ*_0_ of the sisal yarn for N = 100 of 2P-Weibull-LS are 6.11 and 145 MPa, respectively. In contrast, in the 2P-Weibull-ML case, we found that *m_σ_* = 4.47 and *σ*_0_ = 147 MPa. The experimental value obtained in this case was 135 MPa (N = 100 tests).

**Figure 8:**
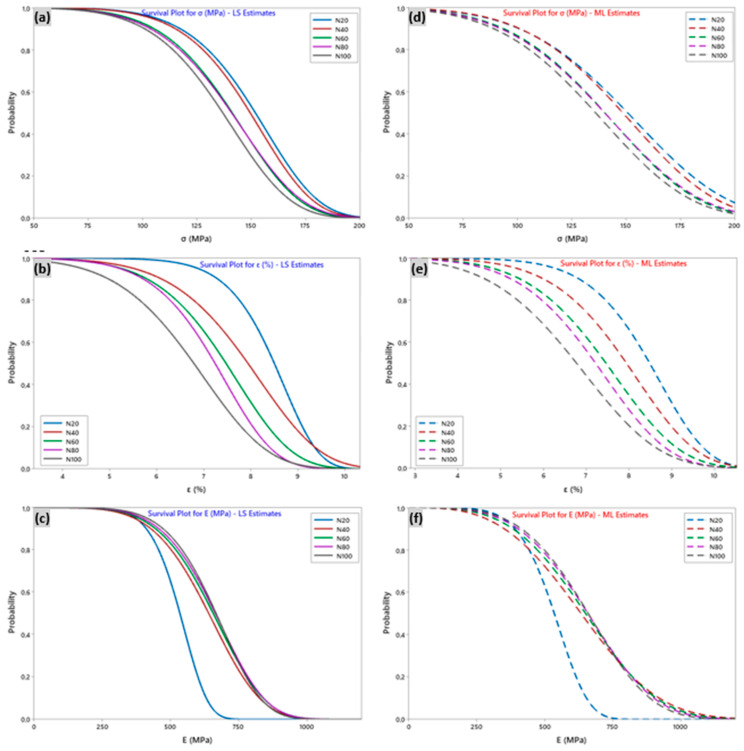
Survival plot for tensile properties with the number of tests N for (**a**–**c**) least square estimation-LS and (**d**–**f**) maximum likelihood-ML estimations.

Using the Minitab software for Weibull-LS and ML analyses, the probability of survival at multiple estimates corresponding to the mechanical properties, including strain, stress, and Young’s modulus, were plotted in Figure 8. For example, the graph in Figure 8a was constructed with the LS approximation of the probability index (Equation (9)) for five pairs of Weibull stress parameters (*m_σ_* = 7.29 and *σ*_0_ = 159, *m_σ_* = 7.2, *m_σ_* = 6.42 and *σ*_0_ = 149, *m_σ_* = 6.18 and *σ*_0_ = 149, *m_σ_* = 6.11 and *σ*_0_ = 145, respectively, for N = 20, 40, 60, 80, and 100 mm). Thus, it can be observed that, when the likelihood *P*(*σ*) = 0.4 in the case of 2P-Weibull-LS, corresponding to a 40% population survival for the wire samples with N = 80 and 100 trials, the stress was evaluated simultaneously as 147 and 143 MPa. However, when the survival rate reached approximately 50%, the lowest values were 137 and 140 MPa for the same number of trials. For tensile strain and stress, *P*(*ε*) = *P*(*σ*) = 0.4, we obtained 7.2% and 143 MPa, respectively. In addition, for N = 100 tests and for N = 80, we obtained *ε* = 7.6%, and *σ* = 147.

**Figure 9:**
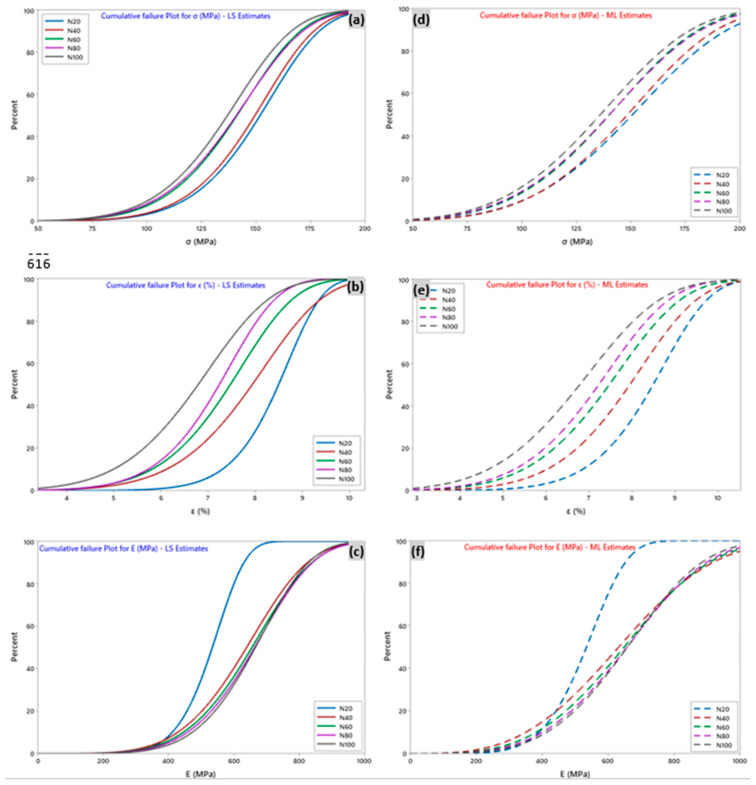
Cumulative failure plot for tensile properties with the number of tests N for (**a**–**c**) least square estimation-LS and (**d**–**f**) maximum likelihood-ML estimations.

The cumulative failure plot (Figure 9) allows us to determine the cumulative probability in % for a yarn element that meets a failure at load levels less than or equal to the specified load level, and thus, to evaluate the reliability of the sisal yarn from its failure. Furthermore, the cumulative failure function is the difference between 1 and the survival function. As an example, based on the data (N = 100 tests) related to the breaking stresses of the yarns, it appears that the probability of a yarn breaking when the stress reaches 165 MPa is about 0.90. This means that we can be 90% sure that the yarn will break at a tensile stress of 165 MPa for the LS estimate. This was similar for the other properties: *E* = 846 MPa and *ε* = 8.3% for the Young’s modulus and strain, respectively. For the ML estimate, the stress, strain, and Young’s modulus values were 172 MPa, 8.5%, and 905 MPa, respectively.

**Figure 10:**
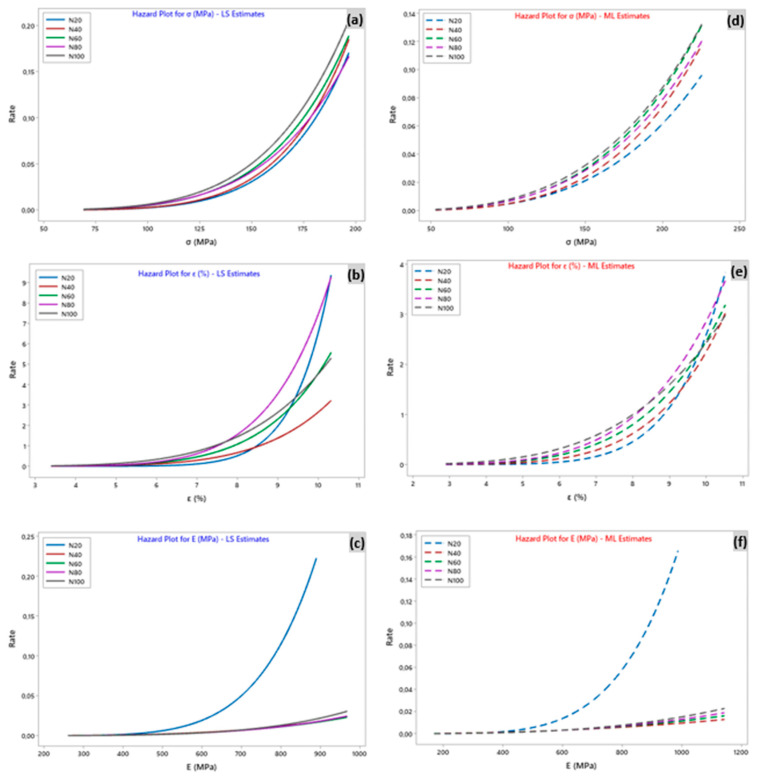
Hazard plot for tensile properties with the number of tests N for (**a**–**c**) least square estimation-LS and (**d**–**f**) maximum likelihood-ML estimations.

There are several methods to determine the hazard function, which represents the probability of a defect as a function of the survival time of the sisal yarn. In the case of sisal yarn, the probability of a defect is a function of the survival time of the yarn. It can be observed that all the curves in the figures (Figure 10) have an increasing exponential trend, which means that the elements have a higher hazard of breaking as the load increases. In general, an increase in hazard occurs at the end of yarn breakage, especially when many fibers break simultaneously, resulting in sudden yarn breakage. The profile of the curve depended on the data, and the model was chosen for the analysis. As an example, Figure 10a is plotted according to the Weibull-LS function with five pairs of shape and scale parameters of the data (*m_σ_* = 7.29 and *σ*_0_ = 159, *m_σ_* = 7.21 and *σ*_0_ = 157, *m_σ_* = 6.42 and *σ*_0_ = 149, *m_σ_* = 6.18 and *σ*_0_ = 149, *m_σ_* = 6.11 and *σ*_0_ = 145 for N = 20, 40, 60, 80, and 100 mm, respectively). This translates into a hazard plot; therefore, the hazard rate increases with increasing loading. Furthermore, it is clear that for the same equivalent load rate of 195 MPa, the hazard rate was reduced when the number of tests N increased (rate = 0.17 for N = 20 up to 0.22 for N = 100). Moreover, for Weibull-ML (Figure 10d), the estimated rate was from 0.09 for N = 20 to 0.13 for N = 100, which is significantly lower than the rate estimated by the Weibull-LS model. The same interpretation applies to the other figures (Figure 10b,c,e,f).

### 4.6. ANOVA Analysis of the Mechanical Properties for Yarn Data

The statistical treatment applied to the data is a method to better understand and analyze the significance of the experiment’s findings. In the current study, the population samples were examined using one-way analysis of variance (ANOVA) based on the mean and distribution of data. This is because there was a large difference between the averages of multiple groups. The two potential hypotheses for the ANOVA technique’s kind of hypothesis test are as follows: The first hypothesis states that all sample means are identical or not statistically distinct from one another. In addition, the number of yarns is a determining factor in the choice of sample. Thus, to best define any mechanical property parameters, Fisher’s test, P, CI, MS, and SS were utilized for ANOVA to establish the impact of the number of tests on the answers.

Table 8 lists all of the results of the one-way analysis of variance (ANOVA) test with a 95% confidence level for the sisal yarn mechanical characteristics (*ε*, *σ*, and *E*) for the various research groups (20, 40, 60, 80, and 100). Due to the *p* value = 0.000 (*p* < 0.001) being below the significance limit (0.05), it is, therefore, not possible to retain the null hypothesis, indicating that averages would be identical.

## 5. Conclusions

In this work, it was evaluated to what extent the number of tests (N) could influence the mechanical characteristics of elementary sisal yarns (tensile stress (*σ*), Young’s modulus (*E*), and strain at break (*ε*)). For this purpose, it was necessary to conduct a series of experimental static tensile tests, which allowed us to determine the mechanical properties for five series of N tests (20, 40, 60, 80, and 100 tests) to then identify and deduce the most efficient number of tests. The main conclusions drawn by this study from the experimental results and Weibull’s law analysis can be summarized as follows:

○ From the tensile tests applied to sisal yarn, it was found that *σ* and *ε* of the yarn decreased with the increase in N from 20 to 80 mm and stabilized from 148 MPa to 138 MPa and from 8.41% to 7.15%. This was followed by a slight decrease in values for N = 100 tests, which produced 135 MPa for stress and 6.70% for strain at break;
○ According to the experimental results, as far as the sisal yarn is concerned, it appears that the best mechanical performance was obtained for N = 100 tests;
○ Moreover, the mechanical properties of the yarns were more consistent with the 2P-Weibull-LS distribution than with the other ML method;
○ Finally, a one-way ANOVA analysis was also employed and revealed that N strongly influenced the sisal yarn mechanical characteristics.

Research results on the tensile properties of sisal yarns have significant practical implications for improving the manufacture of durable ropes and composite structures. They provide essential information for optimizing design, improving mechanical performance, reducing risk, and developing new materials and manufacturing techniques in these fields.

## Figures and Tables

**Table 1:** Material characteristics of sisal yarn used in this work.

	The Number of Fibers	Density (g/cm^3^)	Yarn Cross Section Area (mm^2^)	Tex (g/1000 m)	Surface Twist Angle (◦)
Sisal yarn	70–80	1.433 ± 0.012	4.52 ± 0.43	223 ± 42	10–13°

**Table 2:** Mean, standard deviation, and covariance for different mechanical properties of sisal yarn tested in tensile static tension in this work.

N	Strength (MPa)			Strain (%)			Young Modulus (MPa)		
	Mean	SD	CoV	Mean	SD	CoV	Mean	SD	CoV
20	148	23.85	10.81	8.41	0.91	16.03	528	93	17.72
40	146	25.71	17.49	7.83	1.29	16.43	633	181	28.48
60	139	27.16	19.53	7.37	1.27	17.28	648	169	26.07
80	138	30.67	26.06	7.15	1.21	16.83	657	172	26.06
100	135	29.87	23.97	6.70	1.14	21.02	660	158	23.97

**Table 3:** Summarized mechanical properties data with GL and number of tests of different vegetal yarns from the literature.

Yarn	Strength (MPa)	Strain (%)	Young’s Modulus (MPa)	GL (mm)	Number of Tests	Reference
Jute	52.8 ± 15.23	3.77 ± 0.77	2.28 ± 0.63 GPa	50	25	[34]
Jute	43	7.5	310	50	50	[5]
Jute	117	-	-	100	30	[22]
Jute	248	3.5	-	80	10	[8]
Jute	117	4.39	1978	50	30	[19]
Jute	53.03	3.84	2150	50	10	[35]
Jut	74.8	0.03	2769.7	100	10–15	[15]
Flax	224 ± 45.5	1–3	11,400 ± 2110	500	95	[28]
Flax	271	2.69	10,800	80	30	[20]
Flax	198.1	3.22	5913.6	100	10–15	[15]
Flax	32.7 (N)	1.93	1351 (N)	250	20	[36]
Hemp	124.1	2.94	4236.9	100	10–15	[15]
Hemp	74.86± 6.42	0.277 ± 0.031	1.046 ± 0.172	30	-	[1]
Hemp	449	4.28	11,910	50	20	[37]
Coir	51.8	0.16	322.1	100	10–15	[15]
Sisal	31.5	0.38	85.2	100	10–15	[15]
Sisal	180 ± 25	14.76 ± 1.59	336 ± 184	50	30	[13]
Sisal	148 ± 31	8.37 ± 0.97	556 ± 106	100	30	[13]
Sisal	141 ± 28	6.39 ± 0.59	792 ± 232	150	30	[13]
Sisal	127 ± 24	5.70 ± 0.59	688 ± 175	200	30	[13]
Sisal	122 ± 26	5.00 ± 0.46	616 ± 124	300	30	[13]

**Table 4:** Summary table of test for normality using Kolmogorov–Smirnov test criteria for N100 tests.

	Failure Strength (MPa)	Strain (%)	Young’s Modulus (MPa)
*p*-value	0.025	0.043	0.015
Skewness parameter	1.00585	0.75178	1.04974

**Table 5:** AD and *p*-values estimates for different distributions for N100 tests.

	Strength (MPa)		Strain (%)		Young Modulus (MPa)	
Distribution	AD	*p*	AD	*p*	AD	*p*
Normal	1.474	0.005	0.751	0.049	0.640	0.092
Lognormal	0.374	0.081	0.442	0.083	0.516	0.087
2P-Weibull	2.606	0.110	1.302	0.132	1.694	0.212
3P-Weibull	0.587	0.133	0.434	0.307	0.620	0.109

**Table 6:** AD goodness-of-fit estimates for different distributions.

N	Strength (MPa)				Strain (%)				Young Modulus (MPa)			
	Normal	Weibull	Log-Normal	3P-Weibull	Normal	Weibull	Log-Normal	3P-Weibull	Normal	Weibull	Log-Normal	3P-Weibull
20	1.529	0.921	3.499	1.036	1.255	0.989	2.869	0.835	1.090	0.837	1.923	0.920
40	1.316	0.756	3.402	0.765	0.474	0.484	1.662	0.584	1.389	0.537	2.883	0.628
60	1.085	0.475	2.784	0.860	0.864	0.543	2.358	0.660	1.895	0.482	1.938	0.452
80	1.375	0.503	3.597	0.946	1.721	0.989	4.917	1.252	1.866	0.664	1.814	0.690
100	1.618	0.523	4.400	0.826	0.890	0.551	2.545	0.604	1.771	0.626	1.821	0.637

**Table 7:** Summarized mechanical properties with statistical data of sisal yarns.

N	Strength, *σ* (MPa)					Strain, *ε* (%)					Young Modulus, *E* (GPa)				
	Weibull Statistic-LS														
	Scale	95% CI (Lower, Upper)	Shape	95% CI (Lower, Upper)	R^2^	Scale	95% CI (Lower, Upper)	Shape	95% CI (Lower, Upper)	R^2^	Scale	95% CI (Lower, Upper)	Shape	95% CI (Lower, Upper)	R^2^
20	159	145, 164	7.29	6.17, 9.77	0.900	8.75	8.31, 9.03	12.42	9.83, 14.48	0.910	561	515, 591	7.20	5.42, 9.10	0.944
40	157	147, 162	7.21	6.25, 8.61	0.930	7.34	6.89, 8.66	7.21	5.71, 8.81	0.990	691	630, 729	4.69	4.03, 5.43	0.930
60	149	141, 154	6.42	5.60, 7.41	0.958	7.84	6.51, 8.10	7.45	6.32, 8.41	0.964	706	659, 738	4.86	4.23, 5.52	0.958
80	149	140, 155	6.18	5.32, 7.09	0.957	7.57	6.91, 8.28	8.01	6.98, 9.28	0.946	713	631, 722	5.06	4.61, 6.12	0.964
100	145	142, 150	6.11	5.37, 7.22	0.955	7.20	6.48, 8.54	6.10	5.22, 8.01	0.972	712	629, 725	5.49	4.92, 6.80	0.969
	**Weibull Statistic-ML**														
20	163	147, 179	4.91	3.50, 6.29		8.83	8.37, 9.32	8.77	6.46,11.90		568	525, 613	5.98	4.32, 8.28	
40	160	149, 171	4.69	3.97, 6.06		8.37	7.98, 8.79	6.78	5.36, 8.57		709	639, 786	3.19	2.59, 3.94	
60	151	142, 160	4.66	3.91, 5.54		7.92	7.57, 8.28	6.04	5.02, 7.27		719	667, 775	3.55	2.99, 4.22	
80	151	144, 159	4.52	3.88, 5.27		7.67	7.37, 7.97	5.93	5.07, 6.94		723	680, 769	3.80	3.28, 4.41	
100	147	140, 154	4.47	3.92, 5.12		7.28	6.98, 7.59	4.99	4.32, 5.77		721	685, 759	4.04	3.55, 4.61	

**Table 8:** ANOVA test for strength, strain, and Young’s modulus data of the sisal yarns for 95% CI.

Source	DF	Seq SS	Adj MS	Adj SS	Contribution	F-Value	*p*-Value
A. ANOVA test for ultimate tensile stress (for N = 20 to 100 samples)							
BG	4	6860	1714.9	6860	2.55%	1.93	0.105
WG	295	262,000	888.1	262,000	97.45%		
Total	299	268,860	-	-	100.00%		
S = 29.8016; R-sq = 2.55%; R-sq(adj) = 1.23%; PREESS = 270,897; R-sq(pred) = 0.00%							
B. ANOVA test for strain at failure data (for N = 20 to 100 samples)							
BG	4	71.82	17.955	71.82	12.82%	10.85	0.000
WG	295	488.19	1.655	488.19	87.18%		
Total	299	560.01	-	-	100.00%		
S = 1.28642; R-sq = 12.82%; R-sq(adj) = 11.64%; PREESS = 50.461; R-sq(pred) = 10.10%							
C. ANOVA test for young’s modulus (for N = 20 to 100 samples)							
BG	4	312,564	78,141	312,564	3.57%	2.73	0.029
WG	295	8,434,610	28,592	8,434,610	96.43%		
Total	299	8,747,175	-	-	100.00%		
S = 169.091; R-sq = 3.57%; R-sq(adj) = 2.27%; PREESS = 8,707,691; R-sq(pred) = 0.45%							

BG: between group; WG: within group; DF: degree of freedom; SS: sum of squares; MS: mean square; F: F-test for ANOVA-one way.

## Data Availability

The data presented in this study are available on request from the corresponding author.
